# The Pathogenic Mechanisms of and Novel Therapies for Lamin A/C-Related Dilated Cardiomyopathy Based on Patient-Specific Pluripotent Stem Cell Platforms and Animal Models

**DOI:** 10.3390/ph17081030

**Published:** 2024-08-05

**Authors:** Xin-Yi Wu, Yee-Ki Lee, Yee-Man Lau, Ka-Wing Au, Yiu-Lam Tse, Kwong-Man Ng, Chun-Ka Wong, Hung-Fat Tse

**Affiliations:** 1 Cardiology Division, Department of Medicine, School of Clinical Medicine, Li Ka Shing Faculty of Medicine, The University of Hong Kong, Hong Kong SAR, China; ritaxyw@connect.hku.hk (X.-Y.W.); carol801@hku.hk (Y.-K.L.); vymlau@hku.hk (Y.-M.L.); aukawing@hku.hk (K.-W.A.); yltse2@hku.hk (Y.-L.T.); skykmng@hku.hk (K.-M.N.); wongeck@hku.hk (C.-K.W.); 2 Centre for Stem Cell Translational Biology, Hong Kong SAR, China; 3 Cardiac and Vascular Center, The University of Hong Kong-Shenzhen Hospital, Shenzhen 518053, China; 4 Hong Kong-Guangdong Stem Cell and Regenerative Medicine Research Centre, The University of Hong Kong and Guangzhou Institutes of Biomedicine and Health, Hong Kong SAR, China; 5 Advanced Biomedical Instrumentation Centre, Hong Kong SAR, China; 6 Centre for Regenerative Medicine and Health, Hong Kong Institute of Science & Innovation, Chinese Academy of Sciences, Hong Kong SAR, China

**Keywords:** dilated cardiomyopathy, Lamin A/C, disease models, drug screening

## Abstract

Variants (pathogenic) of the *LMNA* gene are a common cause of familial dilated cardiomyopathy (DCM), which is characterised by early-onset atrioventricular (AV) block, atrial fibrillation and ventricular tachyarrhythmias (VTs), and progressive heart failure. The unstable internal nuclear lamina observed in *LMNA*-related DCM is a consequence of the disassembly of lamins A and C. This suggests that *LMNA* variants produce truncated or alternative forms of protein that alter the nuclear structure and the signalling pathway related to cardiac muscle diseases. To date, the pathogenic mechanisms and phenotypes of *LMNA*-related DCM have been studied using different platforms, such as patient-specific induced pluripotent stem-cell-derived cardiomyocytes (iPSC-CMs) and transgenic mice. In this review, point variants in the *LMNA* gene that cause autosomal dominantly inherited forms of *LMNA*-related DCM are summarised. In addition, potential therapeutic targets based on preclinical studies of *LMNA* variants using transgenic mice and human iPSC-CMs are discussed. They include mitochondria deficiency, variants in nuclear deformation, chromatin remodelling, altered platelet-derived growth factor and ERK1/2-related pathways, and abnormal calcium handling.

## 1. Introduction

**Figure 1:**
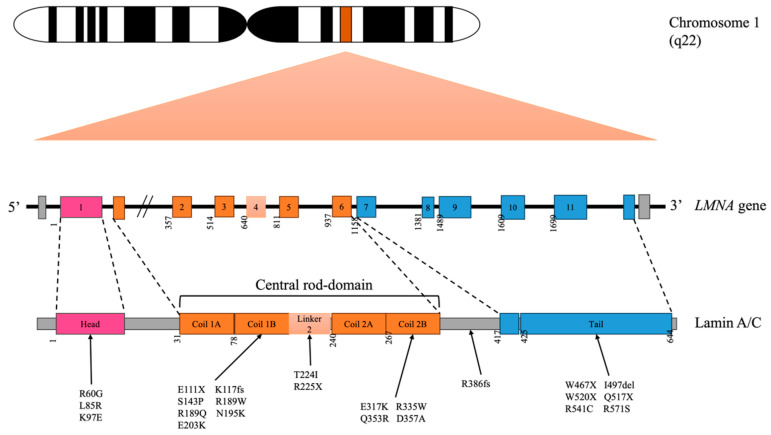
Schematic diagram illustrating the LMNA variants with DCM phenotype in this review.

Lamin A/C-related dilated cardiomyopathy (DCM) is one of the most common inherited cardiomyopathies and is characterised by early-onset atrioventricular (AV) block, supraventricular and ventricular arrhythmias, and progressive heart failure [1]. DCM has been diagnosed with heart failure in the presence of an enlarged left ventricle, left ventricular ejection fraction <45%, left ventricular end diastolic volume index >117%, and fractional shortening <25% on electrocardiography [2,3]. McNally et al. reported that around 30% to 50% of patients with DCM have familial DCM, of whom 40% have genetically driven DCM, including *LMNA*-related DCM. The *LMNA* gene is located on human chromosome 1q21-22 shown in Figure 1, in which alternative splicing into A-type lamin (lamins A and C) contributes to the construction of the nuclear lamina. The unstable internal nuclear lamina observed in lamin A/C-related DCM is associated with the disassembly of lamins A and C, suggesting that *LMNA* variants produce truncated or alternative forms of protein, altering the nuclear structure and signalling pathway related to cardiac muscle diseases [4,5]. The nuclear lamina links to the inner nuclear membrane; is associated with nuclear pore complexes; and organises chromatin to support the nucleus, interface the cytoskeleton and nucleus, and regulate nuclear activities [6,7]. Today, over 450 variants have been identified in the *LMNA* gene that result in a wide range of inherited human “laminopathies”, including familial partial lipodystrophy of the Dunnigan variety, puberty-onset generalised lipodystrophy, limb-girdle muscular dystrophy, restrictive dermopathy, Emery–Dreifuss muscular dystrophy, Hutchison–Gilford progeria syndrome, and dilated cardiomyopathy [4]. It is vital to develop novel therapies for LMNA A/C-related DCM as it is a progressive disease that has a poorer prognosis than other forms of inherited DCM [8,9] and can ultimately lead to end-stage heart failure requiring heart transplantation [10,11].

## 2. Lamin A/C Variants Related to DCM

Up to 50% of patients with DCM have familial DCM, with identifiable variants detected in 40% [3]. As shown in Table 1, various pathogenic variants in the *LMNA* gene have been reported in patients with LMNA A/C-related DCM [1,12,13,14,15,16]. They include missense variant or nonsense variant to an altered protein or a truncated protein due to insertion, deletion, substitution, or frameshift variant at different domains of the *LMNA* gene. A premature stop codon that appears in nonsense variant at the *LMNA* gene yields a truncated lamin A, whereas a change to one amino acid in a missense variant of the *LMNA* gene produces defective lamin A/C protein with an unstable and misfolding structure [17,18]. Missense variants at the N-terminal head of lamin A/C, such as p.R60G, p.E82K, p.L85R, and p.K97E, have been associated with the early onset of DCM (at age 28–40 years) and a high incidence of AV block [16,19]. The missense *LMNA* p.S143P variant located in coil 1b of the rod domain and interacting with lamin B was reported to account for approximately 7% of all DCM cases and up to 28% of familial DCM in eastern and southern Finland [20]. Similar to other missense variants, the *LMNA* p.S143P variant is associated with early-onset progressive AV block, atrial fibrillation, and ventricular tachycardia in severe DCM phenotypes [20]. Other *LMNA* variants at coil 1b, such as the p.E111K, p.K117fs, p.R189W, p.R190W, p.N195K, and p.E203K variants, also present with varying degrees of AV block but a later onset of DCM between the ages of 39 and 64 years [15,16,21,22,23]. The nonsense *LMNA* R225X variant, which exchanges a single base (c.675C>T) in the linker 2 of lamin A/C, has been associated with DCM and a high incidence of conduction disturbance and ventricular tachyarrhythmias [18,24,25]. Interestingly, some variants at coil 2b of the *LMNA* gene, such as E317K, D357A, and R335W, have been reported to result in a less severe form of DCM [12,14,26]. On the contrary, the *LMNA* p.Q353R variant at the same coil of the *LMNA* gene is associated with malignant phenotypes with end-stage heart failure and life-threatening arrhythmias [27]. Finally, those with variants p.R386fsX21, p.W467X, p.Q517X, p.W520 and P.I497-E536de, and the p.R541 variant located in the C-terminal tail, usually present with a more severe DCM phenotype with progressive heart failure [14,28].

The majority of variants at the linker or the edges of lamin A/C manifest at a younger age (11–40 years) [13,28,31,32], while those at the end of the C-terminal tail cause later-onset DCM [16]. Variants at coil 1b and 2b of the central rod domain induce later-onset DCM with a moderate phenotype, and variants at the other remaining sites are associated with early-onset AV block.

## 3. Mechanisms of Lamin A/C-Related DCM

The unstable internal nuclear lamina observed in LMNA-related DCM arises from the disassembly of lamins A and C, suggesting that LMNA variants produce truncated or misfolded forms of protein that alter the nuclear structure and the signalling pathway related to cardiomyopathy [4,5]. Nevertheless, the pathophysiological mechanisms remain unclear. A better understanding of the underlying disease mechanisms in different *LMNA* variants should provide important insight for the development of novel therapeutic approaches. Different disease modelling platforms of LMNA-related DCM are critical for discovering the potential disease mechanisms and the genes altered by the mutated LMNA gene. Different animal models for inherited cardiomyopathies have been created via genetic manipulation. Knock-in animals that express mutant proteins can be generated to help determine the pathophysiology of particular cardiomyopathies and explore new therapeutic strategies in vivo [33]. In vitro cellular models, such as patient-specific induced pluripotent stem-cell-derived cardiomyocyte (iPSC-CMs) technology, provide a novel means to model human cardiomyopathies to investigate pathogenic mechanisms as well as screen novel drug therapies [34]. Table 2 shows different potential pathophysiological mechanisms of *LMNA*-related DCM that have been determined using these in vitro and in vivo platforms.

### 3.1. Mouse Models of LMNA A/C-Related DCM

Both small and large animal models have been developed to study DCM including *Drosophila*, *mouse,* and *primate* models. To easily observe the difference in *LMNA* variants, *Drosophila* models were genetically modified, and the variants were found to differ in nuclear formation, muscle size, adipose tissue, and life span [45]. An *LMNA* c.357-2A>G *Primate* model was generated to observe the similarity of cardiac function to that of humans and provide a preclinical translational platform [46]. Since the *mouse* model is more similar to humans, has a relatively short life span (1–2 years), and is easily manipulated, a number of studies have generated an *LMNA* A/C variant mouse model to mimic the phenotype of *LMNA* A/C-related DCM and to determine the pathogenic role of the *LMNA* gene in DCM shown on Table 3. Studies have deleted or introduced a lamin A/C variant in a mouse model to investigate the pathological role of lamin A/C deficiency in dilated cardiomyopathy, for instance, using *LMNA*-null, p.N195K, p.H222P, and p.R541C mice [39,44,47,48]. Various transgenic mouse models have been adopted to further study the mechanisms and potential treatment of laminopathy-related DCM, such as *LMNA* p.E82K, p.R225X, and p.Q353R transgenic mice [27,37,39,43].

*LMNA* null mice were the first model generated, which were used to identify the absence of the *LMNA*-gene-triggered truncated lamin A product and cardiac function abnormalities in juvenile homozygous LMNA^−/−^ mice (3–6 weeks) by altering the nuclear structure, organisation and function, as well as nuclear–cytoskeletal interaction [35,48,49]. In contrast, an heterozygous LMNA^+/−^-induced lamin A/C insufficiency model caused cardiac conduction defects in juvenile mice and DCM in older adults [50]. Another study introduced the cardiac-specific expression of FLAG-tagged human lamin A in homozygous *LMNA*^−/−^ mice to investigate the role of lamin A in cardiac function [36]. In this study, reintroduction of lamin A improved cardiac function in homozygous *LMNA*^−/−^ mice with the restoration of cardiac contractility, enhanced left ventricular systolic function, and a reduction in the abnormally prolonged PR interval [36].

In addition to the *LMNA* null variant, the *LMNA* p.N195K variant that presents in patients with DCM was introduced in mice to study the pathological characteristics, mechanisms, and potential treatment of *LMNA*-related DCM [47,51]. In these studies, the heterozygous *LMNA*^+/N195K^ mice exhibited no DCM phenotype, but homozygous *LMNA*^N195K/N195K^ mice showed symptoms of DCM with conduction diseases, similar to the *LMNA* null variant mice [51]. Although the above phenomenon in LMNA p.N195K mice differed from that in human patients, the homozygous mice exhibited a DCM phenotype similar to that in human patients with the same variant [16,47,51]. Similarly, in *LMNA* p.N195K variant mice, the disease phenotype of laminopathy or dilated cardiomyopathy was not observed in the heterozygous *LMNA* p.H222P variant mice; nonetheless a severe disease phenotype of *LMNA*-related DCM was evident when homozygote mice reached adulthood [40]. Prior studies showed that a *LMNA* p.H222P homozygous variant altered Lamin A/C localisation in heart and muscle and was associated with abnormal heterochromatin distribution and sarcomere organisation [39]. Moreover, typical DCM phenotypes were observed in homozygous *LMNA*^H222P/H222P^ mutant mice with AV conduction defects and progressive LV dysfunction. Finally, another *LMNA* c.1621C>T/p.R541C variant was introduced into mice to study DCM [44]. An *LMNA* p.R541C variant located at the N-terminal Ig-like domain interacted with lamin A/C and other lamina protein but did not affect polymerisation of the lamin filament or nuclear body. In this study, heterozygous mice exhibited no DCM phenotype, but homozygous mice showed a cardiomyopathy phenotype with mitochondrial defeats.

Instead of knockout/in mice, transgenic mice gene with a human *LMNA* variant were also established to mimic human DCM with cardiac conduction disorders to investigate the molecular mechanisms of *LMNA*-variant-induced DCM and the impact of different treatment approaches. In the study of the *LMNA* p.E82K variant, irregular mitochondria, sarcoplasmic reticulum, and nuclei were found in the transgenic mice who exhibited DCM symptoms with activated Fas and mitochondrial pathway [37]. Another transgenic *LMNA* p.R225X variant was found to be lethal in homozygous mice with decreased postnatal weight and survival; heterozygote mice exhibited fibrosis of the AV node and cardiomyocyte apoptosis with left ventricular dysfunction [43]. The increased expression of extracellular matrix (ECM) genes in *LMNA* p.R225X heterozygous mice resulted in decreased expression of cardiac-conduction-related genes. Cai and et al. suggested that the increased AV node fibrotic region and cardiac dysfunction were induced by unregulated ECM genes, including *Itgb3*, *Itgb2*, *Fn1*, and *Col2a*, and downregulated cardiac-conduction-related genes, including *Kcnj2* and *Kcnj3*. Finally, they determined that the left ventricular function of *LMNA* p.R225X heterozygous mice improved after swimming excise compared with that of sedentary mice. As well as investigating the enriched genes in *LMNA*-related DCM, the pathology of lamin A/C deficiency in DCM was studied using *LMNA* transgenic mice with missense variant c.1058A>G, p. Q354R. The *LMNA* Q353R heterozygous embryos revealed that the pathogenesis of *LMNA*-related DCM was due to the perinatal lethality of *LMNA* p.Q353R. Enlarged cardiac chambers with thin left ventricular wall were observed in *LMNA* p.Q353R embryonic mouse hearts [27].

Based on studies using LMNA-related DCM mouse models, the phenotypic similarity and mechanisms of *LMNA* variants can be summarised. Overall, nuclear deformation and conduction system abnormalities are evident along with DCM symptoms in these muse models. Based on the studies of the *LMNA* p.N195K and p.H222P variants, which cause no DCM phenotype in heterozygous mice and less severe cardiac dysfunction in homozygous mice, patients that carry variants in coil 1b of the *LMNA* gene exhibit later-onset DCM [39,47]. Indeed, mouse models of variants in coil 1b of *LMNA* gene develop less lethal cardiac function abnormalities including those of the conduction system and cardiac contractility. Although heterozygous p.R225X mice developed early-onset DCM with AV block, similar to the phenotypes of the LMNA-related DCM-related R225X variant, another *LMNA* p.Q353R was lethal in heterozygous transgenic mice but was not observed in patients with this variant. The differences in the age of onset in mice and humans can be explained by the developmental trajectory differences between the mouse and human heart [52]. Although patients with *LMNA*-related DCM carry only one allele of *LMNA* variants and have well-documented clinical phenotypes, a large proportion of *LMNA* mouse models exhibit a DCM phenotype with homozygous mutants. One possibility is that lamin A/C’s biological function or its related interaction might differ between mice and humans, such that the critical signalling pathways are not altered in mice with lamin A/C haploinsufficiency.

### 3.2. Human-Induced Pluripotent Stem-Cell-Derived Cardiomyocyte Models

Various cell types have been developed to study cardiovascular diseases, including cardiomyocytes, fibroblasts, endothelial cells, vascular cells, and perivascular cells. Cardiomyocytes, which constitute 70–85% of the total heart, are the most commonly used model cell type for cardiomyopathy research [53]. Since primary cells are difficult to maintain and have a limited lifespan, hiPSC-CMs, which have prominent advantages, have been developed over the past two decades to remodel cardiomyopathies. hiPSC-CMs provide an ideal and well-developed platform to simulate human cardiomyopathies in vitro and investigate the mechanisms underlying cardiomyopathies and screen new pharmacologic therapies for a specific cardiomyopathy [34]. Patient-specific iPSC-CMs derived from people with disease also provide an unlimited cell source to reproduce and study the human cellular disease phenotype. They can imitate the structure and function of human cardiomyocytes as well as the morphological appearance, structure, proteins, ion channel expression, contractile function, and electrical conductivity [34,54,55].

To date, human iPSC-CMs derived from patients with DCM and different *LMNA* variants, including K117fs, S143P, R225X, Q353R, and R541C, have been generated for disease modelling and drug testing in vitro [34,41,53,56,57,58]. One study that used *LMNA* K117fs iPSC-CMs focused mainly on the arrhythmic phenotype and its related pathways [23], while another study compared *LMNA* p.R541C with knock-in *LMNA* hiPSC-CMs to demonstrate the relationship between laminins and chromatin via the *LMNA* B1-associated domain (LAD) [59]. These studies revealed that an irregular distribution of H3K9me2 through the nuclear periphery and lamin-associated domain regions (LADs) was associated with the occurrence of arrhythmias in *LMNA*-related DCM [23,59]. Moreover, a fragile lamina was observed in *LMNA* p.S143P heterozygous hiPSC-CMs with increased nucleo-plasmic lamin A, cellular stress, abnormal calcium loading and arrythmia [38]. Heat shock proteins (Hsps) such as Hsps 90, 70, and 60 were elevated and regulated cardiac function under conditions of stress, suggesting they may have cardioprotective and/or proapoptotic effects in DCM cell lines. *LMNA* variants p.Q353R and p.R225X have been generated in both transgenic mice and iPSC-CMs to elucidate the underlying mechanism of *LMNA*-related DCM [27,41]. In an *LMNA* p.Q353R iPSC-CM study, a distorted and irregular nuclear envelope was observed. Similarly, *LMNA* p.R225X patient-specific iPSCs were derived to explore the response of cardiomyocytes to medical treatments aimed at reducing the apoptotic phenotype and improving functional abnormalities [18]. Accordingly, a distorted nuclear shape, upregulated proapoptotic markers, and abnormalities in contractility and calcium influx were revealed in *LMNA*-DCM hiPSC-CMs. Subsequently, numerous drugs discovered through this ideal platform, including the PDGFRB inhibitor, TT-10, TRPV4 inhibitors (HC-067047 & RN-1734), and PTC124 and MEK1/2 inhibitors (U0126 and selumetinib), were found to have ameliorative effects in *LMNA*-DCM hiPSC-CMs.

Correspondingly, the potential pathologies of *LMNA* variants were studied in terms of enriched genes, nuclear envelopes, and intracellular calcium, which correlated with lamin A/C expression through recapitulating the human cardiomyocytes. Nonetheless there are distinct differences between the phenotypes of iPSC-CMs and mature cardiomyocytes. Cardiomyocyte-specific genes such as troponin, α-actinin, and both α- and β-myosin heavy chai, are expressed in hiPSC-CMs as well as immature cardiomyocyte-specific genes such as connexin 45 and smooth muscle actin [34]. The electromechanics of calcium handling and metabolism also differ between iPSC-CMs and mature cardiomyocytes. For instance, the conduction velocity of iPSC-CMs is 10–20 cm/s, compared with 60 cm/s for mature cardiomyocytes; iPSC-CMs show less-synchronised Ca^2+^ transients than adult cardiomyocytes, and most of the energy for iPSC-CMs is derived from glycolysis rather than fatty-acid β-oxidation [34,60]. Nevertheless, since iPSC-CMs have similar calcium loading properties, nuclear structures, and contraction and action potential profiles to mature cardiomyocytes, the mechanisms and treatment of lamin A/C haploinsufficiency in *LMNA*-related DCM can be modelled in vitro using an iPSC-CM-based platform [61].

Moreover, the potential application of an in vitro hiPSC-CM platform for disease modelling has been limited by the lack of full maturation as well as the potential interactions between different types of cardiomyocytes and other cell types. Recent advances in the development of a 2D co-culturing system of hiPSC-CMs with fibroblasts and epithelial cells, or even 3D culturing of hiPSC-CMs in the form of cardiac organoids, should further enhance the maturation of hiPSC-CMs in vitro as well as provide more comprehensive modelling of cardiac physiology in terms of heart structures, contractile function, ATP generation, and metabolism [62,63,64,65]. Indeed, recent studies have demonstrated the feasibility of generating both ventricular-lineage and atrial-lineage organoids and developing an automated computational approach to compare the phenotypic differences between wildtype and mutant iPSC-cardiac organoids [62]. That study revealed that the phenotypic differences between wildtype and NKX2.5 variants that contribute to the chamber developmental defects could be attributed not only to the gene downregulation in ventricular cardiomyocytes but also differences in cell-type distribution that could not be modelled using a conventional 2D in vitro hiPSC-CM platform [62]. Furthermore, the generation of a 3D cardiac organoid enabled long-term in vitro culture to investigate the development of delayed cardiac phenotypes and pathogenic changes, such as the fibrosis observed in different inherited cardiomyopathies, e.g., Duchenne muscular dystrophy (DMD) [63].

Although these 3D culturing models can overcome some of the limitations of the existing 2D culture methods, they fail to simulate the fully mature phenotypes and functional characteristics of adult human cardiomyocytes. Ongoing efforts to optimise the differentiation and culture protocols for hiPSC cardiac organoids, aiming to further enhance their maturity and the complex interactions between different types of cardiomyocytes and noncardiomyocyte cell types, e.g., cardiac fibroblasts [64], should further improve the in vitro modelling of the different forms of inherited cardiomyopathy, including LMNA-related cardiomyopathy.

### 3.3. Potential Therapeutic Targets

**Figure 2:**
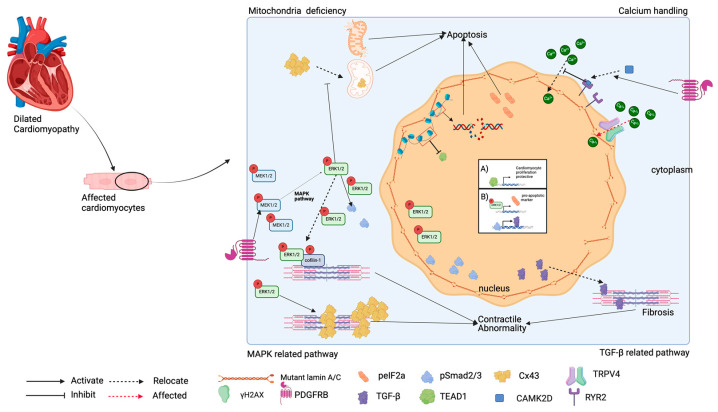
Schematic diagram of signalling pathway in a cardiomyocyte with lamin A/C variant factors such as pERK1/2, pSmad1/2, and TEAD1 affect the gene expression of LMNA mutant cells. Dysregulation of gene expression from mutant alleles causes apoptosis and fibrosis. Also, activation of pMEK1/2 and pERK1/2 disrupts sarcomeres or mitochondria, resulting in contractile dysfunction or apoptosis of LMNA mutants, respectively (shown in A&B). Nonetheless, the signalling pathways in LMNA-mutant-affecting arrhythmias remain unclear.

As shown in Figure 2, the pathogenesis of DCM with conduction system abnormalities in laminopathies is likely multifaceted and includes disrupted chromatin modelling, abnormal activation of mitogen-activated protein kinase (MAPK) and TGF-β-related pathways, and altered calcium loading related to *LMNA* variants.

#### 3.3.1. Mitochondria Deficiency

Studies of *LMNA* p.E82K and p.R541C revealed that laminopathies induce mitochondrial defects [37,44]. A study of the *LMNA* p.E82K variant indicated several proapoptotic factors; caspase-3, -8, and -9 were activated in *LMNA*-variant mice, which was accompanied by increased FAS and relocalisation of cytochrome c from mitochondria to cytosol [37]. In addition, in a study of *LMNA*-related DCM, mitochondria dysfunction contributed to systolic dysfunction in DCM [66]. Hence, restoration of mitochondrial function may be a potential treatment for *LMNA*-related DCM.

#### 3.3.2. Chromatin Modelling

The alterations in heterochromatin distribution and its specific marker (histone3 lysine 9 dimethylation, H3K9me2), LADs, Hf1b, and TEAD1 have been observed in *LMNA* variant models, suggesting that the *LMNA* gene plays important roles in modifying chromatin and transcription signals [23,27,39,40,44]. A study of homozygous *LMNA*^N195K/N195K^ mice determined that the altered expression of Hf1b, an SP1-related transcription factor (Hf1b/Sp4), in the ventricle affected heart development with consequent conduction defects and ventricular dysfunction [47,67]. Moreover, the interior nuclear binding of H3K9me2 at *LMNA*^R541C/R541C^ mouse nuclei indicated that *LMNA* p.R541C increased heterochromatin-associated gene repression [44]. Indeed, both *LMNA* p.R541C and K117fs iPSC-CMs exhibited an irregular distribution of H3K9me2 with altered chromatin conformation and platelet-derived growth factor (PDGF) pathway, with the resulting increased expressions of PDGFRA and PDGFRB [23,59]. In these studies, the altered lamins-associated domain regions (LADs) were revealed, along with the disruption of H3K9me2 [23,59,68]. Nevertheless, the LADs associated with H3K9me2 were found to be involved in cell survival by regulating the gene expression and CpG methylation in human myocardial tissues [68]. Although the role of epigenetic fibrosis in LMNA-related DCM was not extensively discussed, histone modifiers [69,70] and their regulation of the epithelial-to-mesenchymal transition have been found to promote cardiac fibroblast activation. Accordingly, PDGFRB inhibitors, i.e., crenolanib and sunitinib, were shown to be therapeutic for patients with *LMNA*-related DCM [23].

In addition to the distribution of H3K9me2, studies revealed irregular heterochromatin distribution due to insufficient TEAD1 transcription. A study with *LMNA* p.Q353R mice suggested that the irregular transcription of the TEA domain transcription factor 1 (TEAD1) is linked to the disassembly of lamin A/C and the deformation of muscle structure, with the consequent formatting of poor sarcomeres and nuclear blebs [27]. The role of TEAD1 was further investigated by performing single-cell assays for transposase-accessible chromatin. They revealed a positive relationship between the expression level of TEAD1 and cardiomyocyte maturation and structural development [27,71]. Importantly, TEAD1 was responsible for contractile dysfunction in *LMNA* p.Q353R hiPSC-CMs since the contraction abnormalities were rescued by treatment with TT-10, an activator of YES-associated (YAP) TEAD activity [27].

#### 3.3.3. MAPK-Related Pathway

The activation of the pERK1/2-activated MAPK pathway has been proposed as facilitating abnormal cell proliferation, apoptosis, and the stress response to deformed nuclei in *LMNA*-related DCM [72]. Prior studies in *LMNA* null variant mice revealed that nuclear–desmin interactions may be related to pERK1/2 and Cx43 interactions, as well as responsible for DCM being induced by lamin A deficiency [35,36]. Furthermore, the pMEK1 and pERK1/2 in *LMNA* mutant mice, enriched via electrical stimulation, revealed that cellular apoptosis might be activated via the MEK1/ERK1/2 pathway [18]. Moreover, the abnormal localisation of desmin and gap junction protein Cx43 have been described in homozygous *LMNA*N195K/N195K mice, similar to that in *LMNA* null mice [16,36]. Upregulated pERK1/2, and it phosphorylated cofilin-1, in the heart in *LMNA* variants was found to be associated with myocardial dysfunction [39,40]. Preclinical studies demonstrated that ERK inhibitor (PD98059) and JNK inhibitor (SP600125) protected *LMNA* p.H222P homozygous mutant mice against cardiac contractility dysfunction and cardiac fibrosis [73]. The activation of phosphorylated extracellular-signal-regulated protein kinases 1 and 2 (pERK1/2) was also observed in LMNA p.S143P iPSC-CMs [38]. Moreover, the increased ER stress exhibited in *LMNA* p.S143P mutant mice was associated with the upregulation of pERK1/2 and increased DNA breaks. The administration of MAPK inhibitors (U0126 and AZD6244) was also shown to attenuate the apoptotic effect mediated by electrical stimulation in p.R225X iPSC-CMs [18]. These observations indicated that the inhibition of MAPK could be a therapeutic target for patients with LMNA-related DCM.

#### 3.3.4. TGF-β-Related Pathway

The nuclear deformation exhibited in *LMNA*-mutated hiPSC-CMs and mice has been proposed as the mechanism of cardiac apoptosis or fibrosis due to the enriched proapoptotic markers such as DNA breaks, peIF2α and γH2AX, or fibrosis markers including TGF-β and pSmad 2/3 [38,39,40,74]. Prior studies have suggested that the LV dysfunction in *LMNA*-related DCM may be due to the upregulation of transforming growth factor-*β* (TGF-ß) since TGF-ß phosphorylates Smad2/3 in heart-induced fibrosis [39,67]. An approximate 35% apoptosis rate was reported in both human and animal heart failure, and cardiac apoptosis contributes to myocardial cell loss and the loss of cardiac function [75]. Along with cardiac apoptosis, cardiac fibrosis is involved in genetic cardiomyopathies and heart failure with decreased ejection fraction [76]. Nevertheless, the potential therapeutic effect of the inhibition of the TGF-β-related pathway in LMNA-related DCM remains unclear.

#### 3.3.5. Abnormal Calcium Handling

Recent studies showed that the arrhythmic phenotypes of *LMNA*-related DCM arise from an altered PDGF pathway with increased CAMK2D and RYR2 [23]. Observations based on studies with *LMNA* p.S143P and p.R225X iPSC-CMs demonstrated that truncated lamin A/C protein may affect the intracellular calcium level by affecting either the maximum calcium intake or the time of calcium intake and decay [20,41,42,77]. Moreover, alterations to the calcium ryanodine receptor (RYR2, a calcium release channels) have been reported to contribute to intracellular calcium handling and the consequent contractile and conduction function in LMNA-related DCM [23,78,79]. Furthermore, an abnormal calcium influx response was observed due to the activation of stretch-related transient receptor potential vanilloid 4 (TRPV4) channels, mediated by uniaxial stretch in the *LMNA* p.R225X mutant iPSC-CMs. Treatment with TPRV4 inhibitors HC-067047 and RN-1734 decreased calcium loading in *LMNA* p.R225X iPSC-CMs [42,77]. TRPV4 inhibitor (RN1734) may improve systolic function in patients with DCM as a result of reduced calcium overloading in DCM hiPSC-CMs and TRPV4-mediated myofibroblasts [80,81].

## 4. Future Prospectives

The pathophysiology of DCM is characterised as acquired or genetic, and LMNA-related DCM is one of major familial genetic DCMs [10]. According to the AHA, American College of Cardiology, and Heart Failure Society of America Guideline, medical therapy such as ACE inhibitors, angiotensin receptor blockers, beta-blockers, and vasodilators can lower blood pressure and improve blood flow to prevent or treat heart failure and reduce morbidity and mortality in all patients [3,82]. Device therapies, including a biventricular pacemaker implantable cardioverter defibrillator (ICD), have been considered for patients with severe symptoms [10,83]. We believe the well-developed remodelling models can help us to develop potential therapeutic targets including chromatin deficiency, chromatin modelling, MAPK-related pathway, TGF-β related pathway, and abnormal calcium handling.

Accordingly, pilot clinical trials with a MAPK inhibitor are underway (ARRY-371797) in patients with *LMNA*-related DCM. The preliminary results demonstrated improved exercise capacity and decreased cardiac biomarker N-terminal probrain natriuretic peptide after 48 weeks of treatment [73]. Unfortunately, a subsequent large-scale randomised controlled trial investigating the use of ARRY-371797 for MAPK inhibition in patients with *LMNA*-related DCM was prematurely terminated because clinical efficacy could not be achieved (NCT03439514). Studies have revealed therapies that target genetic disorders with long-term efficacy such as PTC 124 and recombinant associated virus (rAAV) [41,84]. Specifically, PTC 124 can induce read-through of the premature stop codon (nonsense variant) and rAAV can replace the mutant gene [85,86]. Since DCM is associated with left-ventricular systolic dysfunction, and calcium plays a vital role in cardiac contraction [87,88,89], the control of intracellular calcium may restore normal contractile function in patients with DCM. Since the activation of TRPV4 channels has also been associated with cardiac fibrosis [80,81,90], the potential therapeutic benefits of TRPV4 channel inhibition to attenuate calcium overloading and myocardial fibrosis warrant future study.

## 5. Summary

In conclusion, *LMNA*-variant-induced abnormal calcium loading and nuclear deformation promote cardiac apoptosis and fibrosis, contributing to DCM. The conduction system disorders, cardiac arrhythmias, and heart failure observed in patients with *LMNA*-related DCM are likely due to multiple pathogenic mechanisms related to different *LMNA* variants, including mitochondrial deficiency, chromatin remodelling, MAPK- and TGF-ß-related signalling pathway activations, and abnormal calcium handling. The improved understanding of the pathogenic mechanisms of human laminopathies derived from transgenic mouse models and iPSC platforms provides novel insight for development of therapeutic targets and treatment approaches for *LMNA*-related DCM.

## Figures and Tables

**Table 1:** Phenotypic differences in patients with DCM.

Domain	Variant	Codon	Type of Variant		Onset	CDS	EF (%)
			Missense	Nonsense			
N-terminal head	R60G	188G>C	✓		Early [16]	AVB, bradycardia	N/A
	E82K	244G>A [19]	✓		Early [19]	AVB	N/A
	L85R	254G>T	✓		Early [16]	AF	N/A
	K97E	N/A	✓		Early [15]	AVB	Severe
Coil 1B	E111X	N/A		✓	Late [15]	AVB	Severe
	K117fs	348-349insG		✓	Late [23]	AF, AVB	Normal
	N120Lfs*5	357-2A>G		✓	Late [29]	N/A	Normal
	S143P	427T>C	✓		Late [20]	AF, AVB, bradycardia	Severe
	K171K	513+1G>A		✓	Late [30]	AF, AVB	N/A
	R189W	565C>T	✓		Late [12,21]	AF	Severe
	R190W	N/A	✓		Late [15]	AVB	Severe
	N195K	585G>C	✓		Late [16]	AF	N/A
	E203K	707G>A	✓		Late [24]	AF, AVB	N/A
Linker2	T224I	N/A	✓		Early [12]	AF	Severe
	R225X	675C>T		✓	Early [24] Late [12]	AF, AVB, bradycardia	Moderate
Coil 2B	E317K	949G>A	✓		Late [12,15]	AF, AVB, bradycardia	Moderate
	R335W	1003C>T	✓		Early [14]	AF	Moderate
	Q353R	1058A>G	✓		N/A [27]	N/A	N/A
	D357A	1070A>C	✓		Early [14]	AF, AVB	Moderate
C-terminal tail	R386SfsX21	1157+1G>T		✓	Early [14]	N/A	Severe
	W467X	N/A		✓	Early [12]	AF, AVB	moderate
	I497-E536del	1489-1G>T		✓	Late [14]	AF	Normal
	Q517X	1549C>T		✓	Late [14]	AF, AVB	Normal
	W520X	1560G>A		✓	Late [14]	N/A	N/A
	R541C	1621C>T	✓		Early [13,28]	N/A	Moderate
	R541H	1621G>A	✓		Early [13]	N/A	Severe
	R541G	1621C>G	✓		Early [13]	N/A	Moderate
	R571S	1711A>C	✓		Late [16]	AVB	N/A

Abbreviations: Atrioventricular block (AVB), atrial fibrillation (AF).

**Table 2:** Mechanisms of LMNA-related dilated cardiomyopathy.

*LMNA* Variant	Models	Phenotypes	Mechanisms	Treatment
Null	Mice [35,36]	-Nuclear deformation -Cardiac conduction defects -Cardiac contractility dysfunction -Irregular desmin	Altered nuclear–desmin interaction Altered pERK1/2 ↓ Cx43	FLAG-tagged transgenic human lamin A
p.E82K	Mice [37]	-Nuclear deformation -Abnormal sarcomeres -Mitochondria defects	FAS/mitochondrial-related apoptosis pathway	N/A
p.K117fs	iPSC-CMs [23]	-Arrythmias -Abnormal Ca^2+^ handling -Fragile lamina -Altered heterochromatin distribution	Altered PDGF pathway ***↑*** CAMK2D ***↑*** RYR2 ***↑** PDGRB*	*PDGRB* inhibitors
p.S143P	iPSC-CMs [38]	-Fragile lamina -Cellular stress -Abnormal Ca^2+^ handling -Dysrhythmias	Altered pERK1/2 *↑* peIF2α *↑* *hsp90, hsp70*, hsp 60 *↑* γH2AX	N/A
p.H222P	Mice [39,40]	-Conduction defects -Altered heterochromatin distribution -Disrupted sarcomere organisation	Altered pERK1/2 pathway *↑* pERK1/2 *↑* p-cofilin-1 *↑* TGF-β *↑* pSmad 2/3	ERK inhibitor JNK inhibitor
p.R225X	iPSC-CMs [18,41,42]	-Abnormal Ca^2+^ handling -Nuclear deformation -Cell apoptosis	Altered ERK1/2 & pMEK1	TRPV4 inhibitor PTC124 MEK1/2 inhibitor
	Mice [43]	-Fibrosis in AV node -Cardiac dysfunction	*↑ Itgb3*, *Itgb2*, *Fn1*, *Col2a* ↓ *Kcnj2*, *Kcnj3*	Swimming exercise
p.Q353R	iPSC-CMs [27]	-Deformed nuclei -Reduced sarcomere density	↓ TEAD1	Activator of YES-associated (YAP)-TEAD activity (TT-10)
	Mice [27]	-Poor sarcomere formation -Nuclear deformation		
p.R541C	Mice [44]	-Mitochondria defects -Altered heterochromatin distribution	N/A	N/A

Abbreviations: phospho-extracellular signal regulated kinase 1/2 (pERK1/2), connexin 43 (Cx43), calcium-dependent protein kinase type II delta chain (CAMK2D), ryanodine receptor 2 (RYR2), platelet-derived growth factor receptor beta(PDGRB), phospho-eukaryotic initiation factor 2-alpha (peIF2α), heat shock proteins (Hsps), transforming growth factor-β (TGF-β), mitogen-activated protein kinase kinase 1 (MEK1), integrin subunit beta (Itgb), fibronect 1 (Fn1), collagen type II (col2), potassium inwardly rectifying channel subfamily J (Kcnj), transcriptional enhancer factor TEF-1/TEA domain family member 1 (TEAD1). ↑ increased expression, ↓ decreased expression.

**Table 3:** Summary of mouse models.

Variant	Description	Phenotype Onset		Other Diseases
Knockout mice				
Null	No Lamin A/C	+/−	at 10 weeks	N/A
		−/−	Onset DCM at 4–6 weeks; died by 6–8 weeks	
Knock-in mice				
N195K	Missense variant	+/−	No Phenotype	EDMD
		−/−	Late onset	
H222P	Missense variant	+/−	No Phenotype	EDMD
		−/−	Onset at 2 months in males	
			Later onset in females	
R541C	Missense variant	+/−	N/A	EDMD
		−/−	Onset at 6 months	
Transgenic mice				
E82K	Missense variant	Not indicated	Onset at 2 months	N/A
R225X	Nonsense variant	+/−	Onset at 6–8 months	N/A
		−/−	Lethal in neonates, died by 12 days	
Q353R	Missense variant	+/−	Perinatally lethal	N/A
		−/−	Cannot be born	

Abbreviations: heterozygous mutant (+/−), homozygous mutant (−/−), Emery–Dreifuss muscular dystrophy (EDMD).

## Data Availability

Data sharing is not applicable.
